# Randomized controlled trial for the efficacy of electroacupuncture in the treatment of urge urinary incontinence: A clinical study protocol

**DOI:** 10.1097/MD.0000000000019315

**Published:** 2020-02-28

**Authors:** Yu Huang, Shuangjin Qi, Xianming Wu, Na Zhi, Ting He, Miaoxian Shen, Shuo Yang, Qian Mo

**Affiliations:** a Guizhou University of Traditional Chinese Medicine; b Department of Acupuncture, the Second Affiliated Hospital of Guizhou University of Traditional Chinese Medicine, Guiyang, Guizhou Province, China.

**Keywords:** clinical trial, electroacupuncture, study protocol, urge urinary incontinence

## Abstract

**Background:**

Despite that the urge urinary incontinence (UUI) is a nonfatal disease, it can lead to anxiety, embarrassment and depression to the patient. UUI is a common public health problem that can significantly affect the quality of life of the patient. Several conservative treatments have been recommended for the treatment of UUI; however, their efficiency remains unclear, leaving the disease without a real effective treatment. The clinical application of acupuncture to treat UUI is currently considered an effective approach despite the limited evidence that support its efficiency. The aim of this study is to assess the efficacy and safety of electroacupuncture therapy in the treatment of UUI.

**Methods and analysis:**

A randomized, parallel, controlled study will be performed. Patients with UUI treated with electroacupuncture group (EA) will compare with the sham-treated sham EA (SA) patients. A total of 100 participants with UUI will be randomly allocated to either the EA or the SA group with a 1:1 ratio. The treatment of UUI patients will performed 3 times per week, for 8 weeks in 30-minute sessions. At the end of the treatment the patients will be followed-up until week 32. The primary outcomes include scores of incontinence questionnaire-short form, the average 24-hour urgency incontinence episodes, and the average 24-hour urge episodes responses from baseline until the 4th, 8th , 24th, and 32nd week. The secondary outcomes included the average 24-hour urine volume and the average 24-hour micturition frequency responses from baseline until the 8th and 32nd week, as well as the change in incontinence quality of life scores from baseline at the 8th and 32nd week. In addition, the degree of satisfaction of the participants undergoing acupuncture treatment will be measured at the 4th and 8th week. The participants’ clinical acupuncture expectations were evaluated at baseline, and the questionnaire for urinary incontinence diagnosis was used to identify stress incontinence, mixed urinary incontinence, and urge incontinence at baseline.

**Discussion:**

This is a randomized, controlled, observer-blinded trial of electroacupuncture treatment for UUI. The results of this trial will provide more evidence on whether electroacupuncture is efficacious for treating UUI.

**Advantage and Disadvantage**

(1) Through previous studies, electroacupuncture for UUI has been proved effective. However, there is still a lack of research on the treatment of UUI with electroacupuncture and sham electroacupuncture.
(2) This trial will assess the efficacy and safety of electroacupuncture in the treatment of acute UUI and will provide high-quality, current evidence for patients and clinicians seeking safe and effective treatments.
(3) In this study, we evaluated the safety of patients and clinical expectation of acupuncture, which was not common in previous studies.
(4) There might be an overestimation of the electroacupuncture effect due to the small sample size of this trial.

## 1 Introduction

According to the International Continence Society (ICS), urge urinary incontinence (UUI) is defined as the complaint of involuntary loss of urine associated with urgency.^[1,2]^ It is characterized by a sudden compelling desire to pass urine that is difficult to defer.^[3]^ UUI often leads to a severely degraded quality of life due to the associated symptoms such as urgency, frequent urination, and urinary incontinence. In addition, it can lead to psychological problems such as depression and anxiety.^[4]^ UUI affects millions of men and women worldwide, which raises huge economic and social burden.^[5]^ Epidemiological investigation have shown that the prevalence of UUI is between 1. 5% and 36. 4%,^[5,6]^ and the incidence increases with age.^[7]^ It is closely related to gender, race, age, BMI, history of mental illness (including depression), pelvic organ prolapse,^[8]^ lifestyle, and other complications such as high blood pressure, diabetes, cancer, lung disease, heart disease, arthritis, and stroke.^[9]^ The association between weight and the UUI subtype in young/middle-aged women was reported to increase 6-fold when the body mass index was greater than 35 kg/m^2^.^[10]^

Currently, the guidelines^[11]^ for UUI treatment recommend behavior therapy as the first-line therapy, such as bladder training, electrical stimulation, pelvic floor muscle training, and biofeedback therapy. In addition, lifestyle changes include the management of caffeine intake, weight, fluid and diet. Drugs are considered the second-line therapy for UUI, which include oxybutynin, Propiverine, Solifenacin, Tolterodine, Trospium.^[11]^ The third-line therapy includes surgery treatment, botulinum toxin type A injection, the use of 5-hydroxytryptamine receptor blockers, and neuromodulation. The currently recommended treatment is a combination of behavioral therapy and medication. However, a study^[12]^ have shown that behavioral therapy often takes weeks or months to achieve satisfactory results, while compliance is not high in most patients. In addition, it has been shown that drug therapy may lead to some side effects, such as dry mouth, which is the most common side effect, as well as blurred vision, constipation, fatigue and cognitive impairment.^[13]^ Surgery, on the other hand, is also associated with certain adverse reactions.^[14,15]^ Therefore, the clinical application of the current UUI treatments remain limited due to these complications.

Acupuncture is a valuable heritage in the Chinese culture and is considered a scientific tradition. It has the advantage of remarkable curative effects, simple operation procedure, low price and few side effects. Many studies have recently attempted to use acupuncture for UUI treatment and adjuvant therapy, and a large number of clinical studies have reported beneficial clinical effects of acupuncture in improving urinary incontinence and urinalysis in UUI patients.^[16–29]^ However, the evidence remain weak due to the lack of long-term follow-up periods, insufficient sample size, and low quality of related studies, which raises the need for more clinical trials to prove that acupuncture may be an optional treatment for UUI in the future. Therefore, we designed this trial to further assess the efficacy of electroacupuncture for treating UUI. The results of this trial will provide a more scientific evidence for a safe, effective and easy-to-promote treatment for UUI in the future.

## 2 Methods

### 2.1 Study design

**Figure 1:**
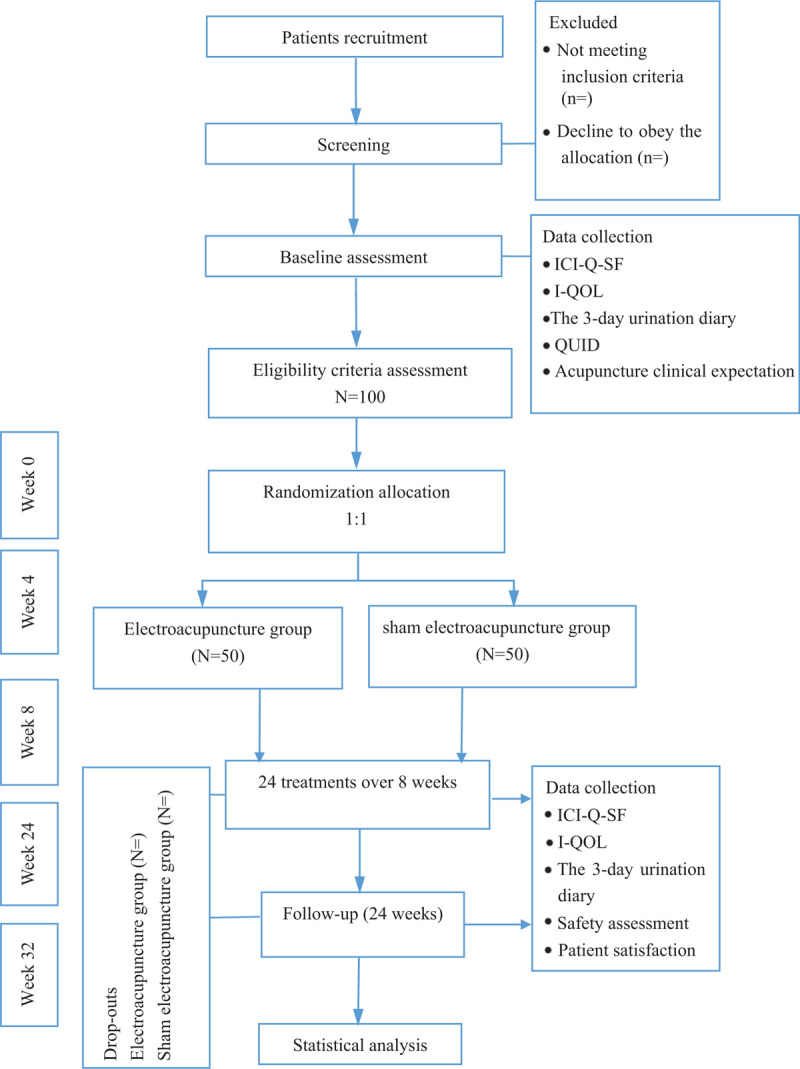
Flowchart of the study design.

This is a randomized, controlled, observer-blinded trial that included 2 parallel groups with a 1:1 allocation ratio. Figure 1 shows the study flowchart.

### 2.2 Participants and recruitment

A total of 100 participants will be recruited between January 2020 and March 2021 through the Second Affiliated Hospital of Guizhou University of Traditional Chinese Medicine website or via public posters. A researcher is responsible for the recruitment and screening of participants, and a urologist is responsible for the diagnosis. A 2-week baseline assessment will be conducted by the researcher, which will be responsible for assessing and recording the participants’ baseline status. After signing a written informed consent, eligible participants will be assigned with random numbers generated by a third party.

### 2.3 Randomization and allocation concealment

The third party will be responsible for randomly grouping the participants and is strictly prohibited to participate in the treatment and data collection during the process. The random grouping is produced using the statistical product and service solutions 22. 0 (SPSS) software, and the final group assignments will be sealed in opaque envelopes. All the envelopes were numbered in the order of the sequence numbers of enrolled cases entering the clinical trial. Hence, the group assignment will be sealed inside and the sequence numbers will be printed on the outside of the opaque envelope. In addition, the acupuncturist will transfer the information of the information of random number and group assignments to each patient by phone or email.

### 2.4 Blinding

Owing to the particularity of clinical acupuncture and moxibustion, it is impossible to use a strict standard blind method for evaluation. Thus, a third party that is blind to the grouping condition carried out the effect evaluation. The efficacy evaluator, the operator and the statistician will be carried out by independent researchers. Subjects, efficacy evaluators, and statistical analysts will be all blinded.

## 3 Participants

### 3.1 Inclusion criteria

In this trial, only patients meeting all of the following criteria will be included:

(1) The diagnosis of UUI is made by a urologist.
(2) The age of the patient is 18 to 75 years.
(3) Frequent urination, urgency, and urinary incontinence. Although the patient knows the intention of urination, it is difficult to control urination.
(4) No drug treatment during the study and no acupuncture treatment within 1 month prior to the study.
(5) Not participating in other clinical studies within 2 weeks prior to this study.
(6) Course of disease at least 3 months.
(7) Patient's vital signs are stable, and is conscious enough to be examined and treated.
(8) Signed an informed consent form to volunteer in the study.

### 3.2 Exclusion criteria

(1) Patients with a history of botulinum toxin infection of the bladder or pelvic muscles in the past year.
(2) Patients with urinary tract infections or vaginal infections during the study period.
(3) Patients undergoing implantable pelvic stimulator therapy during the study period.
(4) Electrostimulation applied to patients in the pelvic region, back, and leg areas during the study period.
(5) The use of drugs that can influence the outcomes before or during the study.
(6) Patients participating in other clinical research related to gynecology, urinary system or kidney function.
(7) Participants with coagulopathy or taking anticoagulants.
(8) Patients with severe heart, liver or kidney damage, patients during pregnancy or lactation, or patients who are generally in poor condition and are unable to cooperate.
(9) Participants with a pacemaker, patients severely afraid of needles, and those fainting or with metal allergy.

## 4 Intervention

### 4.1 The electroacupuncture group (EA)

Acupuncturists with more than 2 years of clinical experience, who have obtained the Chinese licensed physician certificate, are responsible for performing the acupuncture. The choice of intervention plan and acupoint is based on the expert's experience and based on previous studies.^[21,30–32]^ The acupoints of Ciliao (BLadder [BL]32), Zhongliao (BL33) and Huiyang (BL35) will be used (Table 1). All acupoints will be localized according to the name and location of acupoints drafted in 2006 by the National Standard of the People's Republic of China (GB/T 12346–2006). The electric stimulators (SDZ-V electroacupuncture apparatus), and stainless-steel sterilized needles (Huatuo disposable acupuncture needle; 0. 3 mm×50 mm/0. 3 mm×75 mm), were all purchased from the Suzhou Medical Appliance Factory of China. The electric stimulators will apply to the EA and to the sham electroacupuncture group (SA). During the treatment, participants will lie in the prone position, and the acupuncturists will use cotton balls soaked with 75% alcohol to sterilize the skin at the acupuncture site. After skin disinfection, sterile stainless-steel needles will be inserted into the selected acupuncture points. The needles are inserted approximately 50 to 60 mm into the skin with an angle of 60° in an inferomedial direction for BL32/BL33 and a slightly superolateral direction for BL35. After the insertion of the needle, for all needles perform small, equal manipulations of twirling, lifting, and thrusting to reach de qi (these sensations are composed of numbness, soreness, heaviness and swelling), it is considered to be an important component of acupuncture and moxibustion.^[33]^ Paired electrodes from the electroacupuncture apparatus were laterally connected to the needles’ handles of the bilateral BL32, BL33 and BL35. Acupuncture points are continuously stimulated with 100 Hz wave and a 1 to 5 mA electric current for 30 minutes. It is best to make the skin around the acupoint tremble slightly without pain. Participants were treated 3 times a week (every other day) for 8 weeks, with a total of 24 times, and 24 weeks of follow-up after treatment. The fixing pads are placed on the acupuncture points to keep the participants in a blind state.

**Table 1:**
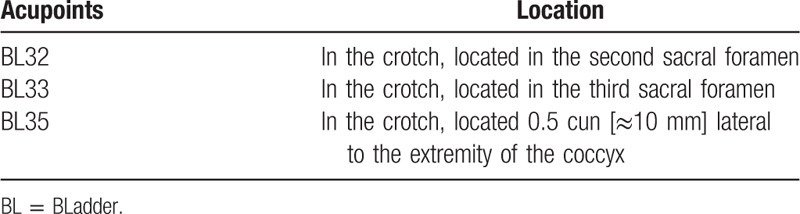
Locations of the acupoints.

### 4.2 The SA group

**Figure 2:**
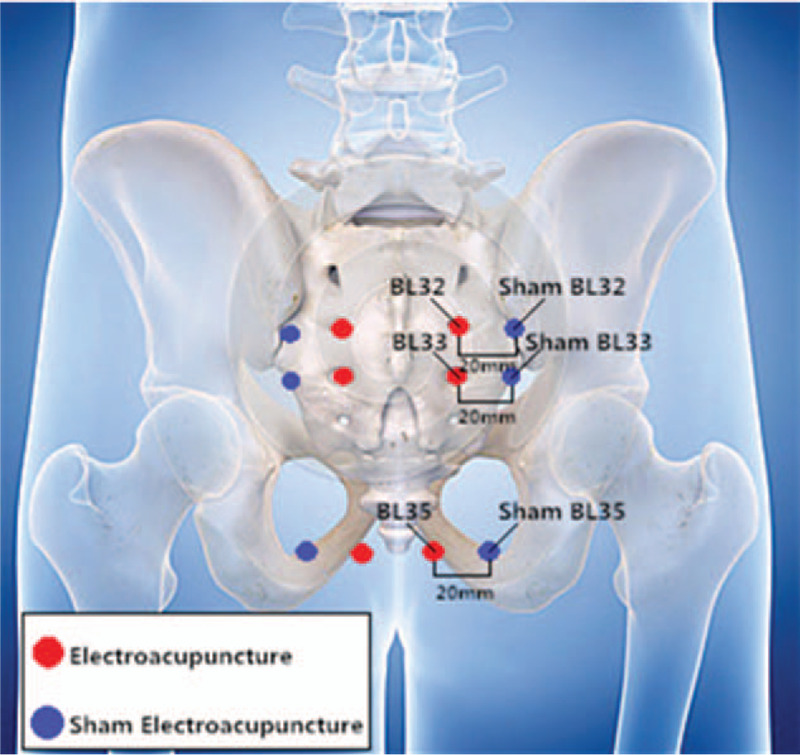
Diagram of the acupoints and non-acupoints (the picture part comes from the internet).

The selected non-meridional and non-acupoint points are 20 mm from the level of BL32, BL33 and BL35, and the selected acupoint are away from the second lateral line of the Bladder Meridian (Fig. 2). During the treatment, participants will lie in the prone position, the skin is disinfected as in the EA group, and the fixation pad is attached to the acupuncture point. A 1.5-inch blunt needle (from Suzhou Medical Appliance Factory of China) is used to puncture through the fixed pad into the skin surface, then evenly twisted and turned, however, without piercing the skin. Paired electrodes from the electroacupuncture apparatus were laterally connected to the needles’ handles of the bilateral sham acupoints. The dedicated power supply cord is cut off, but the appearance is as usual; that is, the electroacupuncture indicator is on, but is actually not powered on. The frequency is 5 Hz and a current intensity of 0.1 to 0.5 mA. The treatment time, waveform and SDZ-V electroacupuncture apparatus are the same as those used for the EA group. The patients are told that it an effective slight current input will be applied that may not result in a sensation of stimulation. The treatment of the 2 groups of participants will be carried out separately in different rooms to avoid communication between the participants. At the end of the trial, participants were given 10 times free compensatory treatments.

## 5 Outcomes

### 5.1 Primary outcomes

a. The primary outcome measure is the score change in incontinence questionnaire-short form (ICI-Q-SF).
  Questionnaire ICI-Q-SF, scores ranging from 0 to 21, with higher scores indicating greater impairment, are compared with the baseline at the 4th (during treatment), 8th (after treatment), 24th (the first follow-up), and 32nd (the second follow-up) weeks of the study.
  ICI-Q-SF is the world's first universal incontinence assessment scale.^[34]^ which is mainly used to assess the frequency of incontinence, the causes of incontinence, and the impact of incontinence on the quality of life. Participants answered 4 questions related to symptoms based on their urination experience in the last week before the test. This includes the number of urine leaks, the amount of urine leakage, the impact of urine leakage on daily life, and when it occurs. The answers are recorded as 0 to 5 points, 0 to 6 points, 0 to 10 points, and the total score between 0 and 21 points. There is 1 unscored question, and the scale Cronbach's alpha coefficient was 0.92.^[34]^ The table is recommended as grade A by the Canadian urological association and the international urinary incontinence advisory committee, and as grade B by the European urological association. It is also recommended for the quantification of the severity of urinary incontinence by the UK's national institute of health and clinical excellence.
b. The change in the average 24-hour urgency incontinence episodes, compared with the baseline and at the 4th, 8th, 24th and 32nd week.
  The 3-day urination diary is another valid, reliable and sensitive measure for patients with UUI. It has been translated into several languages, including Chinese, and is widely used in clinical records to indicate the number of urination during the day, nocturia, urgency, and urgency incontinence, as well as the amount of urine per urination and the amount of drinking water.^[35]^
c. The change in the average 24-hour urge episodes, compared with the baseline and at the 4th, 8th, 24th, and 32nd week.

### 5.2 Secondary outcomes

(1) The change in the average 24-hour urine volume, compared with the baseline and at the 8th and 32nd week.
(2) The change in the average 24-hour micturition frequency compared with baseline and at the 8th and 32nd week.
(3) The change in incontinence quality of life (I-QOL) scores, compared with the baseline and at the 8th and 32nd week.
  The I-QOL questionnaire is based on the satisfaction of participants on the improved quality of life associated with urination. The satisfaction of patients with the treatment was investigated through 3 aspects, namely behavioral limitations, psychological influence and social barriers. The higher the score, the better is the patient's quality of life.
(4) Score of questionnaire for urinary incontinence diagnosis (QUID) at baseline.
  The English questionnaire QUID developed by Bradley^[36]^ et al was used as a tool to diagnose the type of urinary incontinence (SUI, UUI, MUI). The questionnaire includes 6 questions that are mainly focused on the conditions under which patients leak urine and the frequency of urine leakage. The first 3 questions are about SUI and the last 3 are about UUI. Each question has 6 options, each with a score of 0 to 5, and the final total score (0 - 30 points) is obtained by adding the scores of each question. Points on each of SUI and UUI can reach a maximum of 15 points. If the scores of the first 3 questions sum to ≥4, this could be considered as SUI diagnosis. Likewise, if the score of the last 3 questions is ≥6 can be considered UUI diagnosis. On the other hand, if the score of the first 3 questions is ≥4 and that of the last 3 questions is ≥6, the patient is diagnosed as MUI. Because the urgency incontinence and the stress urinary incontinence are easily confused, QUID is used as a mediator for diagnosing urinary incontinence. (Table 2)

**Table 2:**
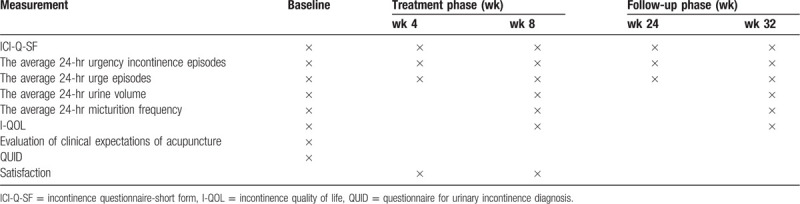
The data measurements at different time points.

### 5.3 Safety assessment

For safety assessment, acupuncture-related adverse events such as scalding, fainting, unbearable acupuncture (VAS ≥8 points), severe post-needle pain (VAS ≥4 points, 10 points) lasting more than 2 hours, local hematoma, infection or abscess; and other discomfort symptoms such as fatigue, palpitations, dizziness, headache and insomnia after the pointer is stabbed, will be assessed throughout the study in both groups.

### 5.4 Adverse events

Once an adverse event was recognized, it is followed up until it is resolved or determined to be permanent. The change in the severity of the adverse event is assessed at each follow-up, with an increase in the frequency of follow-up if necessary, as well as its possible relationship with the treatment. In addition, the means and results of the required interventions are also considered.

### 5.5 Sample size calculation

Sample size calculation for difference in response rates was done as described by Ellenberg.^[37]^ According to previous literature,^[38]^ a 40% reduction in incontinent episodes among patients receiving placebo acupuncture and a 59% reduction in incontinent episodes among patients treated with acupuncture for incontinence. We will need to recruit 50 patients per group from the Second Affiliated Hospital of Guizhou University of Traditional Chinese Medicine to obtain 80% power and a significance level of 5% while allowing for a 20% dropout rate.

### 5.6 Data management and monitoring

All investigators that are trained in clinical practice and who meet the standards independently collect the data and evaluate the treatment effects. All data will be recorded using the ResMan Research Manager, and will be saved and managed by the management platform. In principle, unless necessary for monitoring or unless there is an emergency situation, clinical information will not be not released without the consent of the principal investigator. All documentations and electronic versions of the case report form will be preserved in the secure research archives for 10 years at the Second Affiliated Hospital of Guizhou University of Traditional Chinese Medicine and will only be viewed by the research team.

## 6 Statistical methods and analysis

The data are analyzed using the SPSS software, Version 22. 0 (IBM SPSS Statistics, IBM Corp, Somers, New York). The collected data followed the intent processing principle regarding baseline characteristics, which includes all participants assigned to the group. The primary objective of the trial is to evaluate the average 24-hour urge episodes, the average 24-hour urgency incontinence episodes and the score change in the ICI-Q-SF between the groups from baseline to weeks 4,8,24, and 32. The secondary outcomes include the 3-day urination diary(the average 24-hour urine volume and the average 24-hour micturition frequency), QUID and I-QOL. Independent sample *t* test is used for comparison between the 2 groups, and in case of non-normal distribution, non-parametric Wilcoxon statistics is used to test the null hypothesis. Paired *t* test is used for comparisons before and after treatment between the 2 groups. After comparison between the groups, independent sample *t* test is used if homogeneity of variance was met, while non-parametric Wilcoxon rank sum test is used otherwise. The *χ*^2^ test is used to assess the satisfaction and safety of the treatments. A *P* < .05 indicates that a difference is statistically significant.

## 7 Discussion

The analysis of clinical trials of acupuncture treatment in UUI performed during the past 2 decades indicated that there are various methods for acupuncture treatment in UUI. Of these methods, electroacupuncture is the most frequently used, which can effectively improve symptoms such as urgency, frequent urination, and urinary incontinence. However, in clinical trials on UUI treatment with electroacupuncture, only few relevant studies included placebo control treatments,^[38,39]^ while most studies had insufficient clinical sample size^[39]^ and were performed using unclear research methods. Hence, the level of evidence obtained from these studies is not high. In this study, we are plan to compare the EA with a sham EA using standardized clinical research methods and objective quantitative evaluation indicators to verify the efficacy and safety of electroacupuncture on UUI patients. This study provides the scientific basis for a safety, more effective and easy-to-promote treatment of UUI patients in the future.

UUI patients often show urgency of urination, frequency of urination and urinary incontinence. The diagnosis is mainly based on the symptoms of the patients.^[11]^ Therefore, we use the ICI-Q-SF score, average 24-hour urge episodes and average 24-hour urgency incontinence episodes as the primary outcome to evaluate the clinical efficacy of electroacupuncture treatment of UUI. ICI-Q-SF is considered an effective, reliable and sensitive assessment method that is widely used in clinical practice.^[34]^ It is also a relatively simple questionnaire and the scores are easy to assess. Other widely used scales and questionnaires, such as the 3-day urination diary,^[35]^ I-QOL, QUID and the evaluation of clinical expectations of acupuncture, can also play subsidiary role in evaluating the results regarding the effect of acupuncture on improving the symptoms and ameliorating the quality of life of patients with UUI.

Finally, a 24-week follow-up period (9–32 weeks) is established to observe the sustained effect of electroacupuncture on UUI patients after the end of the treatment sessions. However, certain limitations are associated with this study that require to be mentioned. Owing to the characteristics of acupuncture, it is hard to blind the acupuncturists and patients. Therefore, the randomization process is tightly controlled so as to minimize the possible bias between the 2 treatments. In addition, the investigators and data analysts are also blinded. Furthermore, there might be an overestimation of the electroacupuncture effect due to the small sample size of this trial. In summary, this study can lay the foundation to design larger randomized controlled trials in the future. The results will provide more reliable evidence and elucidate the value and effectiveness of electroacupuncture in the treatment of UUI patients.

## 8 Research ethics

The trial was conducted in accordance with the principles of the Declaration of Helsinki, and has been approved by the review boards and ethics committees of the Second Affiliated Hospital of Guizhou University of Traditional Chinese Medicine (Ethics approval number: PY2019075). The trial was registered in Chinese Clinical Trials Registry (Registration number: ChiCTR1900027846). All participants will signed informed consents before participating in the trial.

## Author contributions

**Trial design:** Qian Mo.

**Recruitment patients:** Yu Huang, SuangJin Qi, Ting He, Na Zhi.

**Treatment:** Shuo Yang, Qian Mo.

**Trial analyst and contributed:** XianMing Wu, MiaoXian Shen.

**Project administration:** Qian Mo.

**Writing-original:** Yu Huang, Shuo Yang.

**Writing-review and editing:** Yu Huang, Shuo Yang.

**Writing – original draft:** Yu Huang, Qian Mo.

**Writing – review and editing:** Yu Huang, Qian Mo.

Yu Huang orcid: 0000-0002-9911-4299.
